# Multi-Omics Approaches and Resources for Systems-Level Gene Function Prediction in the Plant Kingdom

**DOI:** 10.3390/plants11192614

**Published:** 2022-10-05

**Authors:** Muhammad-Redha Abdullah-Zawawi, Nisha Govender, Sarahani Harun, Nor Azlan Nor Muhammad, Zamri Zainal, Zeti-Azura Mohamed-Hussein

**Affiliations:** 1 UKM Medical Molecular Biology Institute (UMBI), Universiti Kebangsaan Malaysia, Kuala Lumpur 56000, Malaysia; 2 Institute of System Biology (INBIOSIS), Universiti Kebangsaan Malaysia (UKM), Bangi 43600, Malaysia; 3 Faculty of Science and Technology, Universiti Kebangsaan Malaysia (UKM), Bangi 43600, Malaysia

**Keywords:** computational approaches, functional genomics, metabolomics, systems biology, transcriptomics, plant breeding

## Abstract

In higher plants, the complexity of a system and the components within and among species are rapidly dissected by omics technologies. Multi-omics datasets are integrated to infer and enable a comprehensive understanding of the life processes of organisms of interest. Further, growing open-source datasets coupled with the emergence of high-performance computing and development of computational tools for biological sciences have assisted in silico functional prediction of unknown genes, proteins and metabolites, otherwise known as uncharacterized. The systems biology approach includes data collection and filtration, system modelling, experimentation and the establishment of new hypotheses for experimental validation. Informatics technologies add meaningful sense to the output generated by complex bioinformatics algorithms, which are now freely available in a user-friendly graphical user interface. These resources accentuate gene function prediction at a relatively minimal cost and effort. Herein, we present a comprehensive view of relevant approaches available for system-level gene function prediction in the plant kingdom. Together, the most recent applications and sought-after principles for gene mining are discussed to benefit the plant research community. A realistic tabulation of plant genomic resources is included for a less laborious and accurate candidate gene discovery in basic plant research and improvement strategies.

## 1. Introduction

The plant kingdom is comprised of photosynthetic eukaryotes, mainly green plants. The enormous variations among and within plant populations include the physical forms, reproductive mechanisms, carbon assimilation strategies (photosynthesis metabolisms), growth and development and other factors such as responses against pests and pathogens, stress environments and productivity [1]. Plants are drastically subjected to constant changes that appear invisible to the human eye, otherwise regarded as unknown.

The phenotype accounts for highly flexible differences which result from the genetics (G), environment (E), and genetics by environment interaction (GXE). The deoxyribonucleic acid (DNA) molecule is the central hereditary unit, as the genetic material is passed from one generation to the other. Composed of four different nucleotides (adenine, thymine, cytosine and guanine), DNA carries gene fragments that encode protein molecules, of which protein-encoding genes contribute to a relatively minor portion (2%) of the total genetic material (genome). The major fraction (98%) of the genome is represented by non-coding sequences, which may indirectly participate in the protein-coding gene expression mechanisms and actions. The central dogma of molecular biology maintains genetic integrity at each life cycle via replication (DNA–DNA), reverse-transcription (RNA–DNA), transcription (DNA–RNA) and translation (RNA–protein) [2]. On the other hand, gene regulatory elements (enhancers and silencers), non-coding RNAs such as microRNA (miRNA), small nuclear RNA (snRNA), small nucleolar RNA (snoRNA), long non-coding RNAs (lncRNAs), and Piwi-interacting RNA (piRNA) are explicitly reported to affect gene expression levels, DNA methylation, alternative splicing events, and epigenetics [3,4].

While the study of the entire genetic material of an organism is known as genomics, the landscape of all the elemental genes expressed (transcripts) at a given time/condition is referred as the transcriptome. Transcripts are translated into protein molecules which may undergo further modifications to form small molecules of <15,000 Da (known as metabolites). These catalogues of proteins and metabolites synthesized at a given time/condition are studied in proteomics and metabolomics, respectively. Thus, transcripts, proteins and metabolites are central components driving the complexity of a biological organism. The growing application of various omics technologies has marked a burst of scientific and technological omics-based approaches offering a wealth of plant science information. “Omics” data are either interpreted independently or integrated via multi-omics analysis to understand critical questions in plant-based research [5].

Systems biology approaches (SBA) offer a plethora of virtual modelling systems equipped with *in silico* designs for gene function prediction [6]. Revolutionized by high-throughput omics technologies, SBA offers a vast amount of big data generated at the molecular level [7,8]. In parallel, computational biology has gained importance alongside SBA for dissecting and further improving the biological information of the target organisms *per se* [9,10]. Moving forward, conventional approaches that are dependent on sequence information to predict the putative biological functions (Gene Ontology classification) of a target gene have expanded robustly to accommodate organizational level-function annotations: the structural features of a given sequence, the interaction between the gene product and the cellular entity, and the phenotypic diversity of a population. In recent years, machine learning approaches and deep learning architectures such as feature-based and artificial neural networks (convolutional neural networks (CNNs) and recurrent neural networks) have been massively deployed in plant research [11,12]. The latter was evidently highly advantageous. For example, in *cis*-regulatory element (CRE) prediction, the CNN, in the absence of a priori knowledge on the target location, outperforms conventional k-mer enrichment, expectation maximization and Gibbs sampling methods with a lower false positive rate [13,14,15].

In this review, we highlight the use of multimodal-omics data and outline the most prominent tools employed for gene function prediction in plant research. Open-source databases available for plant-based omics studies are presented. Further, several plant-related case studies in relation to gene discovery and pathway reconstruction using unknown genes are discussed. Lastly, we emphasize and suggest the importance of integrated multi-modal analyses for gene function prediction and identification in both basic and translation plant research.

## 2. The Omics-Platform

### 2.1. Genomics

**Figure 1:**
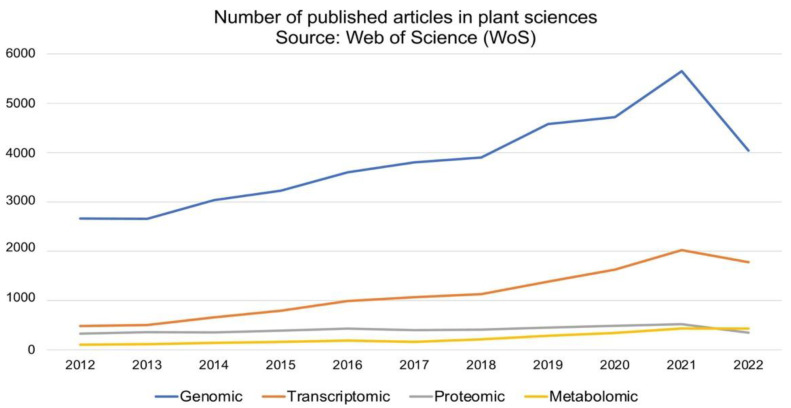
Scholarly omics-related articles published under the plant sciences category from 2012 to 2022. The literature search using Web of Science (https://www.webofknowledge.com) search engine was accessed on 18 September 2022 with Boolean ‘or’ and the following keywords: genomic, genome, transcriptomic, transcriptome, proteomic, proteome, metabolomic and metabolome.

The development and application of next-generation sequencing (NGS) technologies have revolutionized crop improvement strategies primarily through genome exploration and gene discovery [16,17]. Genomics study infers the function and evolutionary history of plants, and with growing NGS technologies such as Illumina, Pacific Biosciences, Beijing Genomic Institute (BGI), Twist Bioscience, 10XGenomics and Oxford Nanopore, the research output (scientific publication) has significantly increased over the last decade (2012 to 2022) (Figure 1). The NGS technologies are indeed robust tools for genome characterization (genome size and genome ploidy level) and genetic variation identification at the genome and/or population level. Genomic datasets are established by means of comprehensive methods which involves the target species’ DNA isolation, sequencing and sequence annotation using bioinformatics tools. Whole-genome sequencing (WGS) requires the entire DNA content of a single organism, while exome sequencing examines the coding DNA sequences (exons) of a genome. Another technique, namely genotype by sequencing (GBS), is a combinatorial technique that employs restriction enzymes to select single nucleotide polymorphisms (SNP) within a population. Epigenomics targets the gene-regulating components such as DNA methylation [18,19].

The decreasing cost of genome sequencing has led to a deluge of plant genome sequences, particularly of agricultural crop sequences [20,21]. Sequencing price varies by the experimental designs and each design considers a myriad of technical features, such as number of reads, read length, methodology and technology. The most used methodologies to generate paired end reads in Illumina are Hiseq (100–250 bp) and Miseq (up to 300 bp). The latter has a low throughput and thus is highly recommended for small genomes <20 Mb. Next, PacBio emerged as a third-generation technology for complex genome sequencing of about 2.5–80 kb. The detection principle is based on the nucleotide excitation of a single molecule, and the technology is subjected to high error rates. The MinION by Oxford Nanopore sequences up to 20 Gb and comes with a low cost, portability features and a high error rate, comparatively much higher than PacBio. Another affordable NGS platform is BGISEQ, a forthcoming technology gaining a foothold in Asia. This technology generates single-end and paired-end reads of about 50–100 bp [22,23]. To date, Illumina remains the best quality read-producing technology. The quality of read profiles generated by Illumina can be evaluated in real time, and poor reads are filtered off using various user-friendly applications as follows: FastQC [24], Cutadapt [25], AdapterRemoval [26], Skewer [27], and Trimmomatic [28].

Plant genome assembly is challenged by the genome size, sequence repetitive nature and ploidy level (autoploid and alloploid). For example, a wheat genome of about 17 Gb features three independent sub-genomes [29]. The genome assembly procedure becomes easiest with the availability of a single allele per locus, although that is not the usual case in most plant genomes. In a systemic comparison between plant and vertebrate genomes using the unbiased kmer-based approach, plant genomes showed higher repeat contents [30].

Upon genome assembly, subsequent genome annotation is required to identify functional elements present along the genome sequence [31]. The genome structural annotation or gene predicting process adds biological meaning to the raw sequences and offers fundamental insights into the biology of the target species. However, the genome annotation process for high-quality genome assemblies is often challenged by the gene density and the introns abundantly present in a genome. There are three distinct computational algorithms developed for detecting the coding region; ab initio (intrinsic), evidence-based (extrinsic) and genomic sequence comparison. The ab initio gene finding prediction software includes the hidden Markov models (HMM), conditional random field, support vector machine, and neural networks. Integrating the information from both the content sensor and signal sensor [31,32,33,34,35], the content sensor classifies the DNA sequence as coding or non-coding, whilst the signal sensor identifies specific functional regions (donor or acceptor of splice site) throughout the genome [30]. Ab initio gene predictors, for instance, GenePRIMP [36], SnowyOwl [37], CodingQuarry [38], BRAKER1 [39], MAKER2 [40], MAKER-P [41] and Seqping [42], can thus be used as a pipeline to predict a reliable annotation on the newly sequenced genomes.

The evidence-based method exploits a cost-effective approach in the form of transcriptional evidence by expressed sequence tags (ESTs) or complementary DNA (cDNA) [43]. The genomic sequence comparison identifies the relativity of the content sensor to the sequence of other genomic DNA [44]. Among the notable comparative gene-finding predictors, CONTRAST [44] has a higher accuracy in both exon/gene sensitivity and specificity than any previous year predictors; N-SCAN, TWINSCAN [45] and GENSCAN [46]. The ab initio and genomic sequence comparison methods are somehow less convincing than evidence-based due to automatic prediction based on training datasets and have poor quality in algorithms that often result in errors.

Genome sequence data facilitate comparative genomic studies targeted to infer the functions of unknown genes [47,48], enable reconstruction of metabolic pathways [49,50] and advance the understanding of evolutionary relationships between and among species [51]. Genome annotation is generally performed using sequence similarity search whereby annotated genes which encode proteins are matched with known proteins available in open repositories [48,52]. To date, plant genomic information can be retrieved from public databases such as NCBI [52] and Ensembl Plants [53]. Meanwhile, PlantGDB [54], PLAZA [55], Gramene [56] and Phytozome [57].

#### 2.1.1. Genomic-Assisted Gene Discovery for Crop Improvement

Genomics is the key enabler of the five Gs in crop improvement instruments: (i) genome assembly, (ii) germplasm characterization, (iii) gene function identification, (iv) genomic breeding and (v) gene editing [58]. Crops with established genome assemblies are research-friendly, as the ease of computational analyses is becoming highly feasible. Plant genetic resources play a fundamental role in leveraging maximum genetic gain in a breeding program. Genetic variation under the natural setting offers breeders the basis for selection and further exploitation for crop improvement. Genetic diversity of highly valuable agronomic traits such as yield, yield-related traits, and resistance against biotic and abiotic components are amongst the most widely exploited traits for further modifications [59]. Generally, mining desirable genetic variants for subsequent improvement serves as the underlying principle of crop genetic improvement. Population-level characterization of genetic variation includes the identification of deletions, insertions, transversions, copy numbers and single nucleotide polymorphisms (SNPs). A germplasm collection holds a broad genetic diversity; thus, the accurate characterization of a large-scale germplasm remains challenging. Nevertheless, advances in genotyping and phenotyping technologies have revolutionized genomic breeding (GB) approaches.

Early GB methods were developed using markers specifically associated with genes and the quantitative trait loci governing major effects of a trait per se. Such methods were extensively applied in early GB programs: marker-assisted selection (MAS), marker-assisted backcrossing (MABC) and marker-assisted recurrent selection (MARS) [60]. Later, in the quest for genetic gain and enhanced breeding efficiency, new, improved methods emerged: genome-wide association study (GWAS), expression QTL (eQTL), haplotype-based breeding, forward breeding (FB), genomic selection (GS) and speed breeding (SB) [60,61].

#### 2.1.2. Single Cell Sequencing

A single cell is the basic structural and functional unit of living organisms. The formation and function of higher-level tissues and organs are influenced by the various genetic mechanisms along stimuli at the cellular environment. Cell heterogeneity refers to the diverse cell states formed throughout cell growth (genetic and molecular biological changes). With highly specialized structures and functions, the cells of multicellular organisms share identical genetics and sets of genetic instructions in the translation of a functional organism. Single-cell genomics offers the cell-specific landscape information regarding the organisms’ genetics, capturing the cell physiology dynamics [62].

The discovery of cell-specific transcription, tissue-specific spatial gene expression, the role of cell localization, the binding and activity of transcription factors, and the chromatin and cis-regulatory signatures of a system of interest is now feasible with growing commercial and specialized equipment systems catered toward resolving cell-specific activities. The chromatin accessibility profiling methods such as the DNase 1 hypersensitive site sequencing and assay for transposase-accessible chromatin sequencing (ATAC-seq) measure the chromatin accessibility for plant regulatory DNA across population-level species [63]. The disadvantages of these methods include a tendency to mask the cell-specific and rare events of a target tissue. Alternatively, improved high-cost systems such as the single-cell ATAC seq assays (integrated co-encapsulation or barcoding of individual cells) perform sequencing at the single-cell level [64]. In transcriptional profiling using the scRNA-seq method, the following strategies are most frequently employed: (i) fluorescence activated sorting (FACS), (ii) isolation of nuclei tagged in individual cell types (INTACT) and (iii) laser capture microdissection (LCM). Both FACS and INTACT have restricted use on selected plant species only, whereas the LCM offers a broader application range on a vast number of plant species. In general, these methods lack markers corresponding to the different differentiation states of the cell types [65]. 

The establishment of the Plant Cell Atlas in 2019 officially marked the trajectory of single-cell studies performed by the plant research community. Comprehensive high-resolution plant cell information (nucleic acids, proteins and metabolites) is built and shared among the scientific community [66]. Single-cell RNA sequencing (scRNA-seq) resolves cell-to-cell heterogeneity using high-throughput technologies: Drop-Seq, Chromium, Seq-well, SMART-seq 3 and iCell8 [67]. These methods offer a variety of features, which account for the following factors: (1) the target mRNA region (5′, 3′ or full length), (2) the number of cells, (3) the cell preparation technique (droplets, cell sorting and nanowells), (4) unique molecular identifiers (UMIs)—the mRNA molecule label, (5) cell size, and (6) method availability. In numerous previous studies, scRNA-seq applied on numerous tissues (Arabidopsis, rice, peanut, maize) revealed high heterogeneity, highlighting the expression signatures of cell types and development trajectories [68]. In the conventional RNA-seq method, the bulk information (average gene expression of the sample) is obtained, whereas the scRNA-seq technique consists of pools of information, each corresponding to the different types of cells present in the sample. The cell preparation is rendered as the utmost challenge to obtaining a decent result with accurate interpretations. Optimizing the protoplast isolation is vital, considering the following factors in a typical plant cell: cell density, cell wall thickness, digestion efficacy (influenced by cuticle, lignin, suberin and other deposition), enzyme type and requirement and enzyme digestion time [67,69].

#### 2.1.3. Genome-Wide Association Study (GWAS)

Amongst these methods, GS is the most preferred tool for breeding programs, as the method does not rely on diagnostic markers entirely and the selection is made on the breeding lines evaluated according to genomic-estimated breeding values (GEBV) generated from the genomic-wide marker data sets. Genomic selection (GS) gathers the additive effects of all the genes governing the genetic variance of a given trait. With each independent gene imparting a relatively small effect, the number of genes controlling a single trait may stretch from hundreds to thousands [60,70]. Using a genome-wide marker and phenotype information, the GS method establishes the association between markers and phenotypes from an observed population. A GS analysis was first performed following Fisher’s infinite model, and soon was extended to the genomic best linear unbiased prediction (GBLUP) model. The latter accommodates GXE interactions and thus offers a more accurate prediction [61,71]. Later, the Markov chain, Monte Carlo and Bayesian modelling methods were developed to include non-additive genetic effects such as adverse environmental conditions. In the GS method, machine learning builds a training/reference population of individuals with information of interest (genotype and phenotype) to train prediction models on the test population or selection candidates. The prediction accuracy is affected by training set population size, density/number of the genome-wide markers and the heritability of the trait of interest [72].

Genomics, together with advanced-level genomic tools, open-source genome resources and powerful technologies, have accelerated crop breeding through rapid trait discovery techniques. Proposed 15 years ago, genomics-assisted breeding (GAB) has now expedited a broad range of breeding programs for resistance enhancement against diseases and tolerance improvement against abiotic factors such as submergence, salinity and drought. In rice, the “Improved Samba Mahsuri”, a GAB product, carries the Xa21, xa13, xa5 and xa38 genes governing the bacterial blight (BB) disease (causal pathogen, *Xanthomonas oryzae*) along with Pi-2 and Pi-54, blast disease (causal pathogen, *Magnoporthe oryzae*) resistance genes [73,74,75].

#### 2.1.4. Pan-Genomics

There are about 390 thousand land plant species, and their genomes are highly complicated (highly repetitive DNA content, polyploidy and heterozygosity) and diverse (genome size varying from 60 Mb to 150 Gb). Plant genome changes arise from evolutionary forces that shaped plant speciation and evolution. Pan-genomics, a subset of plant genomic research, is highly suitable for plant species with extensive genetic diversity at the population level. Pan-genomes have been developed for important agricultural crops and model plants such as rice, Arabidopsis, barley, soybean, maize, wheat, tomato, etc. [76]. The key principles of pan-genomics include the comparison of high-quality genomes to provide insights into the collection of core and dispensable genes in a species population. Generally, a single genome or a small number of genomes do not make a good sample in pan-genome construction. Integration of many high-quality genomes is important to obtain comprehensive genetic information of the target population [77]. Genes are designated as the basic units defining a pan-genome. Pan-genome studies are most useful in understanding plants with a wide spectrum of genetic diversity and gene pools. In brief, the pan-genome strategy first establishes a target population of highly diverse individuals. A good selection of representative individuals in the population is reflected by phenotypic diversity, as determined by the phylogenetic relationship among the individuals of the population. Next, a high-quality genome assembly method for long reads is employed using automatic annotation pipelines. The construction approaches available for pan-genome analyses includes the de novo assembly (detects variant types and classifies genes into core and dispensable), iterative assembly (based on a single reference genome), and graph-based assembly strategy (utilizes graphs from a reference genome to represent the diversity and variations). Comprehensive tools and pipelines popularly employed in pan-genome analyses were exhaustively described by Li et al., 2022 [78].

### 2.2. Transcriptomics

A transcriptome is an atlas of RNA transcripts of a tissue, cell or defined specific condition [79]. Using the genome information, a transcriptome is “read” to obtain a comprehensive description of the genes expressed at a given time point. The mapping and quantification of the transcriptional activity are central to transcriptome studies. In the modern era, the transcriptomes are produced either by the microarray [80] or RNA-sequencing (RNA-seq) technology [81]. The latter is preferred by the plant research community due to higher precision in capturing lowly expressed RNAs and isoforms [81]. Comparatively, the RNA-seq technology detects a greater percentage of novel transcripts than the microarray [82,83]. In most transcriptome data analyses, the raw count data are subjected to differentially expressed genes (DEGs) analysis, co-expression network construction and other techniques such as alternative splicing and isoform analysis [84,85]. Both DEG and network analyses are used extensively to discover genes underpinning various biological processes such as plant defense response [86], regulation [87], water stress *JAZ1* in *G. arboreum* [88], desiccation tolerance and drought (such as LEA) in *A. thaliana* seeds [89], cellulose synthase in secondary cell wall synthesis [90] and cell wall-related genes in *A. thaliana* [91].

In 2002, the Gene Expression Omnibus (GEO) repository was first established as an open repository for gene expression data obtained from various platforms such as microarrays, serial analysis of gene expression (SAGE) and other sequence-based data [92]. Since then, the number of open-source gene expression data repositories for various plant species and condition-specific has been on the rise: The Arabidopsis Information Resource (TAIR) [93], TRAVA [94], RiceXPro [95], Transcriptome Encyclopedia of Rice (TENOR) [96], Barley Gene Expression Database (Bex-db) [97], and Plant Stress RNA-Seq Nexus (PSRN) [98] (Table 1).

Transcriptome data relate to the prediction of genome-scale reconstruction from previous studies: the starch biosynthesis of *Manihot esculenta* [99], the light and temperature acclimation in *Arabidopsis thaliana* [100], and the biosynthesis of biotic stress-regulated pathways (i.e., tryptophan, auxin and serotonin) in *Oryza sativa* [101]. High and low levels of mRNA transcription have improved the understanding of the response outcome in the genome, especially those mechanistic associations between the cellular trade-offs and epistatic gene interactions [102,103].

#### Transcriptome-Wide Association Studies: Prediction of Genes Governing Complex traits

Global transcriptional activity measured by the transcriptome-wide association studies (TWAS) offers a fundamental understanding of the spatiotemporal regulation of transcription events in plants [148]. Transcription causes variation, often observed as a collection of events resulting from altered coding sequences. Both mRNA and protein expression are spatial and temporal targets for selecting variations caused by the coding sequences. TWAS unravel endophenotype or variation that is predominantly caused by genetic factors. Such a feature is highly valuable for prioritizing candidate genes governing complex agronomic traits. TWAS was recently proposed as a powerful tool to predict trait-associated gene expression based on GWAS summary data [149]. TWAS, in combination with GWAS, increases the power of detection of unknown genes and offers a selection of prioritized causal genes [150,151]. 

### 2.3. Phenome

For the past decade, plant phenomics has made significant strides with the advancement of imaging and sensor technologies in measuring a wide range of traits or phenotypic variations in response to environmental factors or genetic modifications [152]. Phenomic data aid in the understanding of the pathways that link genotypes to phenotypes and determine the underlying causes of complex events in crop yields and diseases [153]. Gathering relevant phenotypic data across multiple organizational levels is a key step in phenomics, which aims to characterize the full range of phenotypes that can be expected from a given genome. Therefore, plant phenotyping can be stratified as per resolution and dimensionality (from molecular to entire plant) and environments (from lab to field settings) [154]. The phenotyping method has become an outstanding tool for integrating knowledge into producing high-performance cultivars, particularly for breeders seeking to develop higher tolerant cultivars against abiotic and biotic challenges.

Handling high-dimensional phenomics data necessitates advanced computational methods. In plants, both quantitative trait locus (QTL) mapping and high-throughput phenotyping (HTP) are being utilized to identify the underlying genes responsible for the desired phenotypes. The development of NGS techniques has facilitated rapid and cost-effective access to a vast amount of genomic data, allowing QTL mapping-based marker-assisted selection (MAS) to be conducted. QTL mapping relies heavily on high-quality phenomics data. Near-infrared reflectance spectroscopy (NIRS) data composed of phenomics information have been used as predictors to compare its predictive ability with marker data [155]. The phenomics study via NIRS has been shown to achieve promising predictive abilities in crops, including soybean [156], maize [157] and sugarcane [158]. HTP, on the other hand, relies on automated trait analysis in producing phenotypic data, such as imaging techniques. This technique uses computational image-analysis tools to parse images or videos of traits such as root architecture, height, morphology, and photosynthetic status to extract the latter information [159].

### 2.4. Epigenetic Modification

Chromatin is a complex structure consisting of DNA and histone proteins that are susceptible to epigenetic mechanisms, such as DNA methylation, histone tail modifications and methylation mediated by small RNA (e.g., miRNA, piRNA and/or snRNA). Epigenomics is a dynamic process that alters gene regulation activities that cause plant morphology and development to become abnormal due to environmental factors such as biotic and abiotic stress. DNA methylation patterns vary greatly between plant species. Genes and transposable elements (TEs) in angiosperms are typically methylated at CHG and CHH (H = A, C, or T) nucleotides, whereas CG methylation is highly abundant in animals.

Epigenomic studies using high-throughput sequencing methods, such as methylation arrays, chromatin immunoprecipitation sequencing (ChIP-Seq), assay for transposase-accessible chromatin sequencing (ATAC-Seq), reduced-representation bisulfite sequencing (RRBS-Seq), methylated DNA immunoprecipitation sequencing (MeDIP-Seq), and bisulfite sequencing (BS-Seq), have made it feasible to investigate the roles of epigenetic mechanisms and regulatory pathways in plants at a genome-wide scale [160]. Methylation arrays were the first epigenetic technologies developed to study DNA-methylated CpG islands characterized by the presence of cytosine-guanine sequences. However, the use of methylation arrays in plant studies is still limited compared to other sequencing methods and in mammals [161]. Bisulfite sequencing is widely regarded as the gold standard for detecting 5-methyl-cytosine (5mC) due to its ability to sequence the genome at a base-pair level. Other methods, such as MeDIP-Seq and RRBS-Seq, only examine the preselected genomic regions based on the prevalence of CpG content or methylation [162]. Meanwhile, ChIP-Seq is a powerful method used to study the interaction between transcription factors and DNA binding sites and provides additional information about epigenetic modification based on the chromatin structure or histone changes [163]. To date, more than 11,000 ChIP-Seq data series have been deposited in the Gene Expression Omnibus (GEO) database. Others such as the ATAC-Seq database recorded a total of 1880 data series [164]. By using ATAC-Seq, the chromatin accessibility with DNA methylation changes can be determined using hyperactive Tn5 transposase that cleaves the DNA and then inserts sequencing adapters into open chromatin regions [165].

Epigenomic technologies are widely used to identify genes that underpin various functions. For instance, ChIP-Seq analysis was used by Li and colleagues to identify genes involved in the activation and repression of gene regulation in response to abiotic stress. Additionally, ATAC-Seq profiling of accessible chromatin was carried out to investigate the transcriptional regulatory landscape of plant genomes, which appears to be conserved across the root tips of plant species during development [166,167]. Using BS-Seq, Li et al. (2020) [168] discovered that methylated cytosines (mCG) contributed to the difference in methylation levels in drought stress, revealing extensive DNA methylation changes in response to drought. The MeDIP-Seq profiling of olive development also showed differential DNA methylation in secondary metabolism, which is responsible for the quality of olive oil. This finding provides an insight into the significance of the methylation status of olives during the ripening process [169].

#### 2.4.1. Interactomics

Interactomics, the study of interactions between functional elements within an organism is revolutionizing genetic research. The systemic dissection of functional genes riding the phenotype of interest is analyzed by genetic models or networks within and between genetic layers. For example, the genome-wide protein–protein interaction analysis may represent two distinct genetic layers, namely the proteomics and transcriptomics. An interactome study exploits large datasets generated by multi-omics technologies to improve the predictive power in understanding the role of functional elements of a complex biological system [170]. It offers valuable information on the associations between functional elements across multiple biological processes. The functional elements of an interaction network are represented as nodes, whilst the relationships between the nodes are edges. The edges are constructed based on the correlation measurements derived algorithmically from quantitative omics-datasets [171,172].

A rapid research pace in co-expression network construction and analysis using transcriptomes (RNA-seq and microarray generated datasets) may have arisen from the increasingly growing open-source databases. Databases of co-expression datasets include the ATTED-II [130], AraNet [132], GeneMANIA [141] and others, as listed in Table 1. In higher plants, co-expression network analyses have successfully dissected gene function prediction in glucosinolate biosynthesis [173], cell wall biosynthesis [174], transcriptional regulation of hormone biosynthesis [175] and Arabidopsis aliphatic glucosinolate biosynthetic pathway [176].

Protein–protein interaction (PPI) dissects the physical interactions between a group of proteins, ultimately imparting a global understanding of the functional mechanisms of a proteome landscape, domain interactions and motif and site association of complexes [177]. PPI datasets are generated by means of in vivo, in vitro and in silico methods [178]. The in vivo methods such as yeast two-hybrid (Y2H) [179], split ubiquitin system (SUS) [180] and bimolecular fluorescence complementation (BiFC) [181] test the physical interactions between two proteins. Meanwhile, in vitro methods such as affinity purification mass spectrometry (AP-MS) [182] and protein microarrays [183] quantify PPIs and protein activities. Both the in vivo and in vitro methods serve as evidence in PPI network construction. In addition, various computational methods have been developed for PPI prediction: interolog mapping, gene/domain-fusion inference, domain/motif-domain transfer, gene co-expression network and machine learning approaches.

#### 2.4.2. Resources for Plant Protein–Protein Interactions

The number of plant species-specific experimentally validated or predicted PPIs has been growing with the development of new info-centric databases [127]. As such, the PlaPPISite houses comprehensive and high coverage interactomes of 13 different species (zzdlab.com/plappisite/index.php) [127]. The Interacting Proteins (DIP) [120] and 3D interacting domains (3did) [128] databases integrate information from the Protein Data Bank (PDB) for the identification of protein interaction sites [183]. Concerning plant PPI data, the Biological General Repository for Interaction Datasets (BioGRID) [126], Molecular Interaction database (MINT) [129], Biomolecular Interaction Network Database (BIND) [124], Functional Protein Association Networks (STRING) [119] and *Arabidopsis thaliana* Protein Interaction Network (AtPIN) [126] are rendered the most widely employed databases in plant functional studies.

#### 2.4.3. Integrated Multi-Layer Omics Data for Functional Studies in Plant

**Figure 2:**
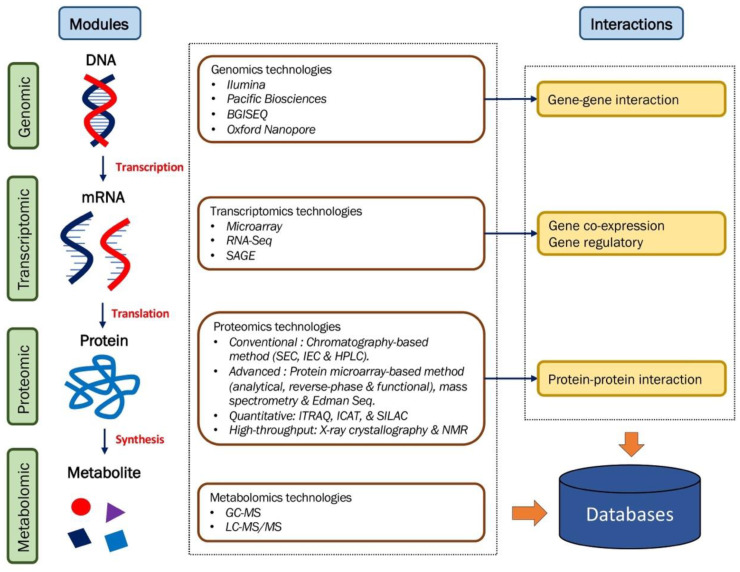
Type of omics datasets. Omics datasets can be divided into two categories: modules and interactions. Module data indicate molecular sequences of biological systems; DNA (genome) transcribed into mRNA (transcriptome), later translated into proteins (proteome), and lastly synthesized into metabolite (metabolome). Interaction data, known as interactomes, represent the relationships of module data generated from respective platforms. The omics resources of the omics technologies and network interactions can be downloaded from the databases.

The functional aspects governing phenotypic diversity are cumulatively driven by the distinct layers of the central dogma. Genetics research, along with integrated multi-omics approaches, has made a major leap forward in gene function prediction and identification. The use of multi-omics datasets established from a single experiment is essential for significant characterization and identification of gene/protein/biomolecules and their putative roles in the biological pathways and processes. The omics-to-interactome relationship using multi-layer omics modules is shown in Figure 2. Integrative omics recruits at least two or more distinct genetic layers, often established from omics technologies.

- Genomics-transcriptomics

Co-expression networks using transcriptome-based analysis have facilitated the characterization of unknown gene functions within the metabolic pathways [171]. A combinatorial analysis of sequence similarity and co-expression identifies conserved co-expression networks across various crop species [184]. With the recent development of co-expression network databases such as PlaNet [136], PhytoNet [145], CoNekT [146], and CoCoCoNet [148], it is now possible to gain insights into the potential causal effect of gene interactions on a trait of interest. In a study by Liu et al. [185], the gene regulatory network (GRN) constructed from transcriptome data elucidated the relationship between transcription factors and target genes via direct interaction. In another study, GRN was utilized to investigate the genome-wide transcriptional response of fruit development [186]. The hub genes (group of tightly associated genes) identified from the co-expression network correlated with TFs, suggesting potential regulatory mechanisms involved in fruit development metabolism [185,186].

- Transcriptomics-proteomics

The integration of genome and transcriptome data may demonstrate the abundance of protein; however, it does not compulsorily correlate with the corresponding mRNA levels [187,188]. This may likely occur when there is a protein synthesis delay at the regulation and post-translational modification process [189], along with other factors such as the density of the ribosomal subunit [190] and physical characteristics of the transcript [191].

In a study which investigated maize leaf development, the correlation between the mRNA and protein abundance was relatively weak during the leaf transition from heterotrophic to autotrophic cells compared to later stages of development [192]. In another study, both proteome and transcriptome data were integrated to understand tomato pericarp ripening [193]. The post-translational mechanism occurred during ripening when the protein abundance and mRNA levels showed a weak correlation, in contrast to the early stage of tomato ripening [193]. Integrative methods are effectively deployed to understand plant responses toward biotic and abiotic stresses. Peng and colleagues suggested that several cotton stress-responsive proteins (gigantae protein, α-crystalline heat shock protein, and β-1-pyrroline-5-carboxylate synthetase) regulate the alternative splicing events as the mRNA levels were significantly correlated with protein abundance under salt-stress condition [194]. The alternative splicing event allows the translation of spliced mRNAs (from a single gene) into multiple proteins [195].

### 2.5. Candidate Gene Mining in the Context of Pathway Reconstruction

In plant biology research, gene function identification is primarily challenging as the study requires a large-scale dimension [196]. In *Arabidopsis thaliana*, approximately 27,500 genes that encode proteins were reported in 2013 by The Arabidopsis Information Resource (TAIR), and this number was expected to increase with time. In the same year, 30% of genes were reported to be experimentally validated, compared to only 11% in 2007 [197,198]. Within 50 years of Arabidopsis research, more than 50,000 publications have been released and stored in the TAIR database for data curation, the annotation of newly discovered genes and metabolic pathway refinement [199]. Candidate gene mining in higher plants is much more challenging compared to bacteria due to tissue-level complexity at the cellular level and the lack of functional information about existing annotated gene functions [43].

#### Challenges in Cellular Pathway Reconstruction

Plants synthesize more than a million different types of metabolites [200]. Cellular pathways such as the metabolic, biochemical, and signal transduction of plant function influence the system-level behavior, growth and development processes. Incomplete metabolic pathways from weak annotation necessitate pathway reconstruction [44,201]. The identification of candidate genes in an incomplete metabolic pathway may result in unknown proteins. These unknown proteins could represent a missing enzymatic reaction underpinning a dead-end metabolite. The first step in pathway reconstruction begins with the identification of orthologous genes. Orthologous genes in different species arise from speciation events. Since a common ancestor holds orthologous genes, they retain a similar gene function. Orthologous domains/proteins are retrievable in Clusters of Eukaryotic Orthologous Groups (KOG) via the Clusters of Orthologous Groups (COG) (https://www.ncbi.nlm.nih.gov) [202] and WU-BLAST2 server (dove.embl-heidelberg.de/Blast2), as accessed on 19 August 2022 [203].

## 3. Guilt-by-Association (GBA), a Method for Gene Discovery

**Figure 3:**
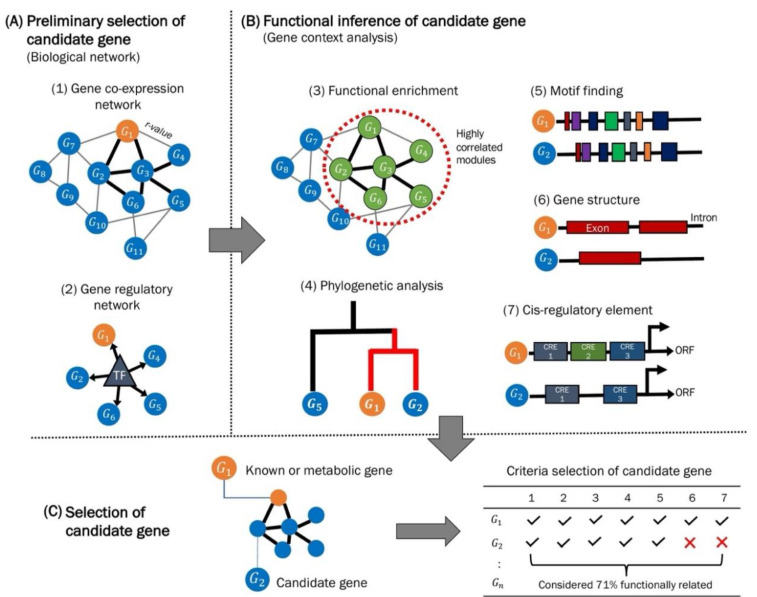
Guilt-by-association techniques for candidate gene discovery. (**A**) Preliminary selection of candidate gene using the biological network, in which the co-functional information of candidate gene with known gene (i.e., metabolic or other functional genes) can be extracted from gene correlation and coregulation/regulatory network (steps 1–2). (**B**) Co-functional information can be inferred by gene context analyses (steps 3–7). Candidate genes will be observed based on enriched in a similar function (step 3), clustered in a monophyletic group (step 4), shared similar distributions of motifs (step 5) and exon/intron structure (step 6), and lastly consist similar CREs (step 7). (**C**) The ranking of the high confidence candidate gene will be observed based on the predicted co-function similarity.

A gene co-expression network (GCN) is a powerful tool to uncover unknown genes based on correlation values computed (gene expression data) among a series of experimental samples/conditions [204]. A candidate gene is assumed to co-function with a partnering gene in the event of correlation, an association measure (Figure 3). The GCN is built by calculating the correlation of mRNA expression levels across samples. The transcripts are represented as nodes connected by either weighted or unweighted correlation values, represented as edges. Unweighted edges imply a binary graph, whilst weighted edges score the different strengths of the edges of a completely connected graph.

In 2000, the “guilt-by-association (GBA)” principle was proposed to unravel the gene function of uncharacterized or hypothetical targets within a functional network [205]. Assuming that two interacting genes or proteins are hypothetically bound to a similar or related cellular function [205,206], the GBA assesses for biological information of a co-expression network such as functional links between genes: plants [207], yeast, and bacteria [208]. Gene co-expression and co-regulation have become a standard technique to identify the function of unknown genes in metabolic pathways [176]. By using mRNA data from RNA-seq/microarray technologies, genes with similar expression profiles are hypothetically presumed to be regulated by a similar transcription factor [209,210].

According to Hansen et al. [207], two genes with similar features (sequence, structure, and expression pattern) may share a similar function. Gene context analysis and gene network study are commonly known as GBA. Genomic evidence using comparative genomics and gene co-expression networks infer the participation of the candidate genes in a similar or related pathway by identifying the possible association with genes of known functions [48,207,211]. According to Osterman and Overbeek [48], the gene context technique ranks candidate genes through multiple assignments. Thus, candidate genes with highly similar contexts are measured as high-confidence genes with potential functional association with known genes [212,213,214,215].

Basically, GCN construction involves three key steps: (i) input data comprising an expression matrix (m = gene across n = conditions) vector with n = dimension (Table 1), (ii) similarity measurement/association measure and gene similarity matrix, and (iii) the threshold value (cutoff correlation value). The association measures are calculated using Pearson’s correlation coefficient (PCC), Spearman’s correlation coefficient (SCC) and others, dependent on the dataset distribution. The gene interaction of GCN is defined as the correlation between genes [211,212,213]. Correlation values that meet the threshold criteria assume significant interactions [212].

The threshold value selection criteria vary for unweighted and weighted GCN [214]. There is no rule of thumb applied for setting the threshold values. Although a soft threshold value (nearing zero) is considered less significant, it compensates for the robustness of a weighted GCN [215]. On the flip side, important genes might be missed out from the network with a highly stringent threshold selection [216]. A hard threshold (r = 0.8 to 1.0) has been shown to be more relevant in studies inferring biological relationships. The validity of the biological information computed based on the GO functional similarity measure increases at r > 0.8 [215]. GCN has been widely applied to Arabidopsis for the identification of genes corresponding to cell wall biosynthetic [90], fatty acid chain [217], photorespiration [218], immune response [219] and other metabolic pathways. In others, a random threshold correlation value was applied in GCN construction: r = 0.7 in GCN of biotic and abiotic attack [220,221] and r = 0.83 for leaf development [222].

In weighted GCN, the strength of the interaction is reflected by the score distribution (0 to 1) [214]. Contrarily, in unweighted GCN, the interaction score is computed by binary values, whereby 0 represents no correlation, and 1 indicates the presence of correlation [214]. The WGCNA [223] and webCEMiTool [224] are freely available computational resources available for weighted GCN construction. Others that feature differential GCN construction include dcanr [225], Ebcoexpress [226], MODA [227], DICER [228], and DiffCoEx [229] and CoExNet [230] for unweighted GCN. The differential GCN infers the causal regulatory changes between sample groups of different conditions [87]. For example, the comparative co-expression of mRNA and lncRNA in *Cleistogenes songorica* under water-deficient conditions identified differentially expressed mRNAs and lncRNA of common TFs families. The function of lncRNAs was identified as drought stress regulation via interaction with miRNAs and protein-coding genes [231].

Recently, another method in GCN, comparative GCN analysis, incorporated gene homology and co-expressed gene information for functional prediction in different plant species [148]. Comparative GCN can be executed by predicting conserved interaction between homolog genes from two or more species. Conserved genes with similar co-expression profiles showed significant biological similarities and differences in Arabidopsis and maize [232]. Obviously, a gene that integrates both the sequence similarity and co-expression profile information provides a better prediction accuracy than independent single-information analysis. The integration of homology gene and correlated gene expression allows useful information on candidate genes to be obtained from the conserved gene modules. The functional annotation could be drawn relatively from model plants to the crops of interest [233,234]. There are various web servers and applications available for co-expression and comparative plant studies; EXPath [134], Plant Network (PlaNet) [136], RED [144], PhytoNet [145], and CoCoCoNet [147]. These tools combine and compare the conserved GCN among plant species, ultimately aiding gene function prediction.

## 4. Predictive Modelling, Artificial Intelligence and Machine Learning Based Methods

The ‘big data’ era in plant sciences offers massive omics-datasets that are extremely large, noisy and heterogeneous in nature. Gene, protein and metabolite prediction using phenotypic datasets from various genotypes under adverse environmental conditions increases the call for scientific approaches that could effectively handle big data with parallel integration of multi-modality phenomics, metabolomics, genomics, proteomics, transcriptomics, etc. [235]. In this context, artificial intelligence (AI) and machine learning (ML) fit perfectly to support the decision-making processes in various plant research areas while accommodating diverse and fragmented datasets: the prediction of genome regions favorable for genetic modifications, modelling the genotype–environment interactions, the dissection of complex plant traits, and the prediction of genome crossover regions. ML comprises algorithms that learn to perform a required task using a given dataset. There are two distinctive types of machine learning: (i) supervised learning, where output prediction is dependent on the input data (training data), and (ii) unsupervised learning, which identifies patterns in an unlabeled dataset [236]. The most common unsupervised methods used in plant research include principal component analysis (PCA), clustering and Autoencoder. The PCA method corrects for data variability by linear transformation of the variables. The clustering method clusters the data observations based on the similarity features. The Autoencoder utilizes artificial neural networks to perform reconstruction using compressed input data to minimize the differences in the original dataset [237,238]. In plant research, the supervised method is much preferred compared to the unsupervised method; nevertheless, the selection of ML methods is largely influenced by data availability and the objective of the analysis [239].

## 5. Conclusions

Viridiplantae is estimated to consist of about several hundred thousand species. With tremendous advances in sequencing technologies and computational tools, genome sequencing and assembly have emerged as important strategies for decoding genetic information of plant species. Undeniably, plant species with decoded genetic information are better placed for manipulation and subsequent improvement in breeding programs. Important crops, primarily food crops such as rice, wheat, sunflower, soybean and many others, have been the species of interest in high-throughput next-generation sequencing (NGS) technology. Leading barriers in the success of elucidating the plant genetic landscape includes the large and inherent complexity of plant genomes attributed by polyploidy, phenotypic variation and heterozygosity factors observed in repetitive sequences, transposable elements (TE), tandem arrays, and ribosomal gene clusters. To date, only <1000 draft plant assemblies have been constructed using the NGS platforms. Nevertheless, new methods are being robustly developed to enhance specificity against the research biological question. Optimized computational algorithms, computational power and sequencing technologies are increasingly catered toward answering specific research questions. The ultimate challenge in gene function prediction involves employing that most appropriate technological tools feasible to the experimenter. With climate change on the chart of global issue, food security requires serious attention in the realm of an ever-growing human population. Plant breeding is the utmost fundamental strategy in crop yield improvement. Conventional breeding programs are being replaced by rapid high-throughput breeding approaches, ultimately to gain better resolution in effective breeding programs. Gene function prediction and identification is a pre-requisite step that informs the design of a plant breeding method. Modern biological research provides comprehensive insights into system-level variation using collated multi-omics tools and integrative system biology approaches. What is the concerted pool of genes, proteins and metabolites underpinning a complex trait? The ease of dissecting the research questions posed here becomes much less with integrative omics analyses which favor high-confidence predictions.

## Figures and Tables

**Table 1:** Plant omics databases, as accessed on 24 August 2022.

Omics Type	Database	Organism	URL	References
Genomics	Plant Genome Database (PlantGDB)	Plants	http://www.plantgdb.org	[54]
	Plant Genome DataBase Japan (PGDBj)	Plants	http://pgdbj.jp/?ln=en	[104]
	National Center for Biotechnology Information (NCBI)	Various	https://www.ncbi.nlm.nih.gov	[52]
	Ensembl Plants	Plants	http://plants.ensembl.org/	[53]
	Phytozome	Plants	https://phytozome.jgi.doe.gov	[57]
	PLAZA	Plants	https://bioinformatics.psb.ugent.be/plaza/	[55]
	Plant Genome and Systems Biology (PGSB PlantsDB)	Plants	http://pgsb.helmholtz-muenchen.de/plant/plantsdb.jsp	[105]
	Chloroplast Genome Database (ChloroplastDB)	Plants	http://chloroplast.cbio.psu.edu/	[106]
	The Solanaceae Genomics Resource (Spud DB)	Potato	http://solanaceae.plantbiology.msu.edu	[107]
	Melon Genome Database (Melonomics)	Melon	https://www.melonomics.net/	[108]
	Maize Genetics and Genomics Database (MaizeGDB)	Maize	https://www.maizegdb.org	[109]
	Rice Annotation Project Database (RAP-DB)	Rice	https://rapdb.dna.affrc.go.jp	[110]
	Rice Genome Annotation Project (RGAP)	Rice	http://rice.plantbiology.msu.edu	[111]
	GrainGenes	Wheat, Barley, rye, oat	http://wheat.pw.usda.gov/GG3/	[112]
	SoyBase	Soy	Soybase.org	[113]
	Genome Database for Rosaceae (GDR)	Rosaceae plants	https://www.rosaceae.org/	[114]
	Brassica Database (BRAD)	Brassica plants	http://brassicadb.org/brad/	[115]
Transcriptomics	Gene Expression Omnibus (GEO)	Various	https://www.ncbi.nlm.nih.gov/geo/	[92]
	AgriSeqDB	Plants	https://expression.latrobe.edu.au/agriseqdb	[116]
	The Bio-Analytic Resource for Plant Biology (BAR)	Plants	http://bar.utoronto.ca	[117]
and	The Arabidopsis Information Resource (TAIR)	Arabidopsis	https://www.arabidopsis.org	[93]
	Transcriptome Variation Analysis (TRAVA)	Arabidopsis	http://travadb.org	[94]
	The Rice Expression Profile Database (RiceXPro)	Rice	https://ricexpro.dna.affrc.go.jp	[95]
	Transcriptome Encycloperdia of Rice (TENOR)	Rice	http://tenor.dna.affrc.go.jp/	[96]
	Barley Gene Expression Database (Bex-db)	Barley	http://barleyflc.dna.affrc.go.jp/hvdb/	[97]
	Plant Stress RNA-seq Nexus (PSRN)	Plants	http://syslab5.nchu.edu.tw	[98]
	Plant microRNA database (PMRD)	Plants	http://bioinformatics.cau.edu.cn/PMRD/	[118]
Interactomics	STRING	Various	https://string-db.org	[119]
	Database of Interacting Proteins (DIP)	Various	http://dip.doe-mbi.ucla.edu	[120]
	Protein–Protein Interaction Database for Maize (PPIM)	Maize	http://comp-sysbio.org/ppim	[121]
	IntAct	Various	https://www.ebi.ac.uk/intact/	[122]
	Oryza sativa Protein–Protein Interaction Network (PRIN)	Rice	http://bis.zju.edu.cn/prin/	[123]
	Biomolecular Interaction Network Database (BIND)	Various	http://bind.ca	[124]
	The Biological General Repository for Interaction Datasets (BioGRID)	Various	https://thebiogrid.org	[125]
	Arabidopsis thaliana Protein Interaction Network (AtPIN)	Arabidopsis	https://atpin.bioinfoguy.net	[126]
	PlaPPISite	Plants	http://zzdlab.com/plappisite/index.php	[127]
	3D interacting domains (3did)	Various	https://3did.irbbarcelona.org	[128]
	Molecular INTeraction database (MINT)	Various	http://mint.bio.uniroma2.it/mint/	[129]
	ATTED-II	Plants	http://atted.jp/	[130]
	CressExpress	Arabidopsis	http://cressexpress.org/	[131]
	Arabidopsis Network (AraNet)	Arabidopsis	http://www.inetbio.org/aranet/	[132]
	Co-expressed Biological Processes (CoP)	Plants	http://webs2.kazusa.or.jp/kagiana/cop0911/	[133]
	EXPath	Plants	http://expath.itps.ncku.edu.tw/	[134]
	Plant Omics Data Center (PODC)	Plants	http://bioinf.mind.meiji.ac.jp/podc/	[135]
	Plant Netwrok (PlaNet)	Plants	http://aranet.mpimp-golm.mpg.de/	[136]
	OryzaExpress	Rice	http://plantomics.mind.meiji.ac.jp/OryzaExpress/	[137]
	PlantExpress	Rice, Arabidopsis	http://plantomics.mind.meiji.ac.jp/PlantExpress/	[138]
	Rice Functionally Related Gene Expression Network Database (RiceFREND)	Rice	http://ricefrend.dna.affrc.go.jp/	[139]
	*Vitis vinifera* Co-expression Database (VTCdb)	Grape	http://vtcdb.adelaide.edu.au/	[140]
	GeneMania	Various	http://genemania.org/	[141]
	A Comprehensive Systems-Biology Database (CSB.DB)	Various	http://www.csbdb.de/csbdb/home/databases.html	[142]
	RapaNet	Brassica	http://bioinfo.mju.ac.kr/arraynet/brassica300k/query/	[143]
	Rice Expression Database (RED)	Rice	http://expression.ic4r.org	[144]
	PhytoNet	Various	www.gene2function.de	[145]
	CoNekT	Plants	https://conekt.sbs.ntu.edu.sg	[146]
	CoCoCoNet	Plants	https://milton.cshl.edu/CoCoCoNet	[147]

## Data Availability

Not applicable.
